# Dietary inflammatory potential in depressive symptoms in older Chinese people in Hong Kong: a cohort study

**DOI:** 10.3389/fnut.2026.1831232

**Published:** 2026-06-01

**Authors:** Agassi Chun Wai Wong, Xue Yang, Timothy Chi Yui Kwok, Jean Woo, Yi Su, Jason Chi Shun Leung, Samuel Yeung Shan Wong

**Affiliations:** 1 Jockey Club School of Public Health and Primary Care, Faculty of Medicine, The Chinese University of Hong Kong, Hong Kong SAR, China; 2 Department of Medicine and Therapeutics, Faculty of Medicine, The Chinese University of Hong Kong, Hong Kong SAR, China; 3 Jockey Club Centre for Osteoporosis Care and Control, The Chinese University of Hong Kong, Shatin, Hong Kong SAR, China; 4 Jockey Club Institute of Ageing, The Chinese University of Hong Kong, Shatin, Hong Kong SAR, China; 5 Key Laboratory of Molecular Epidemiology of Hunan Province, School of Medicine, Hunan Normal University, Changsha, Hunan, China

**Keywords:** depressive symptoms, diet, dietary inflammatory index, geriatric, mental health

## Abstract

**Purpose:**

Depression is a highly prevalent mental disorder that is associated with severe comorbidity. As individuals continue to age, the adverse effects of depression may worsen. Emerging studies from Western cultures have indicated that the elevated Dietary Inflammatory potential in the everyday diet might be associated with depression, but little is known about the Asian Chinese population.

**Methods:**

A sample of 3,740 elderly Chinese subjects from the Hong Kong Mr and Ms Os project was followed for 7 years. To assess changes in depressive symptoms, the Geriatric Depression Scale (GDS) was employed, and the baseline food frequency questionnaire (FFQ) was used to calculate the Dietary Inflammatory Index (DII). The Generalized Linear Mixed Model (GLMM) was used to investigate the longitudinal effects of DII on depressive symptoms. Inverse probability weighting was used to address attrition bias.

**Results:**

Significant associations were observed between DII and depressive symptoms. Higher DII was associated with higher depressive symptoms (β = 0.228 per unit increase) across both sexes, particularly in females. A similar observation was observed when comparing lower DII vs. higher DII by tertile.

**Conclusion:**

This study identified the positive associations between dietary inflammatory potential and depressive symptoms in the Chinese population. Further exploration of the sex difference is warranted. Limitations include a single dietary assessment and substantial attrition.

## Background

Depression is a common mental health disorder that imposes a significant disease burden. In older adults, depression is linked to disability, including cognitive impairment, dementia, and increased mortality (1), and has a complex etiology (2) that has been associated with biological factors such as inflammation, potentially the primary cause (3, 4). The negative impacts of depression may worsen with age (5). According to the World Health Organization (WHO), about 14% of adults aged 60 and over suffer from a mental disorder (6). A recent meta-analysis reported that the worldwide prevalence of major depression among the elderly is 13.3%, with higher rates in females than in males (7). In Hong Kong, the prevalence of depression among the elderly has been documented to range from 4.7% to 8.3% (8, 9). Additionally, the burden of depression is projected to increase by 10% in China by 2025 (10). Therefore, examining modifiable factors is essential to reduce the growing health impact. A previous review reported that elevated inflammatory markers, such as interleukin 6 (IL-6) and C-reactive protein (CRP), were associated with the development of depressive symptoms (11). Furthermore, a recent meta-analysis showed that depressed patients were more likely to have been exposed to low-grade inflammation and elevated CRP levels (12), suggesting that inflammation could be a key factor in causing depression.

An emerging area of interest is the role of nutrition in depression. Nutritional factors are known to influence inflammatory status (1) and are modifiable. Various nutrients possess pro-inflammatory and anti-inflammatory properties. The Dietary Inflammatory Index (DII) was developed to assess overall dietary inflammatory potential (2) and is associated with inflammatory markers. For instance, a cross-sectional study found that a higher DII score indicated a more pro-inflammatory diet and was associated with depression in individuals under 65 (3), but not in older adults. A recent meta-analysis of eight longitudinal and nine cross-sectional studies suggested that dietary inflammation is positively linked to the risk of depression (4). Only one study examined older subjects with a mean age of 65 or above. Moreover, all included studies were conducted in Western cultures. Consequently, the findings were limited to understanding the DII and the influence of Western cultural dietary patterns. Dietary patterns can vary across cultures. For example, food avoidance due to religious beliefs and differences in habitual diets (5) may lead to distinct dietary patterns. We identified only one study in the Chinese middle-aged population (55 or above) (6), which reported that a more inflammatory diet was associated with a higher risk of depression. However, the results were constrained by the cross-sectional design. Therefore, further research is needed to clarify the relationship between DII and depression within the Chinese population.

Furthermore, current evidence on the effects of dietary inflammation across sexes remains inconsistent. For instance, a prospective cohort study found a significant association only among males (7), whereas cross-sectional studies identified associations only among females (8, 9). Sex could influence the relationship between DII and depression. Possible explanations include differences in dietary patterns and the prevalence of depression between males and females.

### The present study

This study aimed to investigate (1) the association of DII score with depressive symptoms among older Chinese adults; and (2) the effects of sex in the association between DII score and depression in a longitudinal study in Hong Kong. Based on the existing evidence, we hypothesized that the DII scores among the elderly population would be positively associated with depressive symptoms, while the association would be moderated by sex.

## Subjects and methods

### Study participants

The present study included participants from the Mr OS and Ms OS cohort in Hong Kong, which is part of a large-scale, multi-ethnic study in Hong Kong, Sweden, and the USA. Mr OS and Ms OS aimed to examine the determinants of osteoporotic fractures in older Chinese adults. In total, 4,000 ambulatory, community-dwelling Hong Kong Chinese adults aged 65 or older were recruited from August 2001 to March 2003 (10). Participants were followed for 14 years, with return visits at the 2nd, 4th, 7th, and 14th years after recruitment. Recruitment was stratified by age (65–69, 70–74, 75 years or above) in equal proportions through advertisements and health talks. Written informed consent was obtained from all participants, and the study was approved by the Clinical Research Ethics Committees of the corresponding author's university. All participants provided informed consent, and those with probable dementia were excluded because they were unable to provide consent. According to a previous study, the risk ratio for depression comparing high DII vs. low DII is 1.16 (11), and Cohen's d is approximately 0.088. Using G^*^power (12), with α = 0.05, 150 participants are needed for power (1 – β) = 0.95.

### Exclusion criteria

Exclusion criteria for the current study were as follows: Subjects who were lost to follow-up (*n* = 2,125), who were outside the pre-defined limits for energy intake [ < 3,347.2 kJ/d (< 800 kcal/d) or > 16,736 kJ/d (>4,000 kcal/d) in males and < 2,092 kJ/d (< 500 kcal/d) or 14,644 kJ/d (>3,500 kcal/d) in females] (*n* = 4), those without outcome measure (*n* = 17) and those who reported with depression using the Chinese Geriatric Depressive Scale (GDS) with a cut-off of 8 or more (10) at baseline (*n* = 243) were excluded from the analysis. After these exclusions, a total of 3,740 (93.5%) participants were followed for 7 years.

### Dietary measurements and DII

A single baseline dietary assessment was conducted using a validated food frequency questionnaire (FFQ) designed for the local population (11), and dietary intake of 280 food items over the past year was measured. This captures daily and weekly food consumption and was administered by trained research assistants. Photographs of common foods and portion sizes were provided in an instruction manual (12). Recognizing that a single assessment assumes relative dietary stability and may not reflect changes over the follow-up period, mean daily nutrient and energy intakes were calculated using food composition tables from the Chinese Medical Sciences Institute and the Centre for Food Safety in Hong Kong (13, 14). When nutrient data were unavailable, tables from the Taiwan Food and Drug Administration and the US Department of Agriculture served as alternatives (15, 16). The Dietary Inflammatory Index (DII) is a scoring system derived from the literature. It has been extensively reviewed and developed for 45 food parameters, analyzing their effects on inflammatory markers such as interleukin (IL)-1 β, IL-4, IL-6, IL-10, C-reactive protein (CRP), and tumor necrosis factor (TNF)-α. The DII score ranges from negative to positive; higher positive values denote a pro-inflammatory diet, whereas lower or negative values suggest an anti-inflammatory diet. Some foods or nutrients with inflammatory effects, such as spices, are rarely consumed in Chinese culture and were therefore excluded. In this study, FFQ-derived dietary data was available for 30 of the 45 food parameters used in calculating the DII. These parameters include energy, protein, carbohydrate, total fat, saturated fat, monounsaturated fatty acids, polyunsaturated fatty acids, fiber, cholesterol, vitamin B12, vitamin B6, folic acid, thiamine, niacin, riboflavin, vitamin A, beta-carotene, vitamin C, vitamin D, vitamin E, iron, magnesium, selenium, zinc, isoflavones, alcohol, caffeine, onion, pepper, and green/black tea. According to previous research, the DII remains valid with 28 to 45 food parameters (17). The mean intake of each parameter was estimated from a global database and transformed into a centered percentile score, multiplied by the inflammation effect score derived from the literature (2). The combined score of the 30 included parameters constituted the overall DII score. Furthermore, the energy-adjusted DII (E-DII) was calculated using the residual method to minimize the influence of total energy intake (18).

### Depressive symptoms

Depressive symptoms were measured using the short version of the Chinese Geriatric Depression Scale (GDS) (10), comprising 15 yes-or-no questions with a score ranging from 0 to 15. The questions relevant to depression, such as motivation, self-image, loss, agitation, and mood, were asked. Of the 15 items, 10 indicated the presence of depression when answered positively, while the rest indicated depression when answered negatively. A higher score indicates greater depressive symptoms. The GDS has been validated among the local population and was found to be highly correlated with clinical depression (19). Scores 0–4 were considered normal, 5–8 indicated mild depression, 9–11 indicated moderate depression, and 12–15 indicated severe depression. A cut-off of 8 or more was used to define the presence of depression in the current study. GDS is an instrument that measures depressive symptoms in older adults, with 81.3% sensitivity, 78.4% specificity (19), and a moderate level of internal consistency (Cronbach's α = 0.63), as tested in the current sample.

### Background variables

Demographics, lifestyles, and medical data were collected using structured questionnaires. Anthropometric measures, including body height (centimeters) were measured using a Holtain Harpenden stadiometer, and weight (kilograms) using a Physician Bean Balance Scale. Body mass index (BMI) (kg/m^2^) was calculated. Age, sex, lifestyle patterns, and chronic illness were shown to be predictors of clinically relevant depressive symptoms (20–22). Based on this evidence, the following available variables in the dataset were selected: age, sex, BMI, diabetes, hypertension, hypothyroidism, marital status, physical activity level, smoking history, alcohol use (yes/no), and self-reported physical activity level were assessed using the Physical Activity Scale of the Elderly (PASE) adapted for the Hong Kong population (11, 23).

### Statistical analysis

Baseline characteristics, lifestyle variables, and depressive symptoms scores among the DII category in quartiles were shown as mean [standard deviation (SD)] for quantitative variables and frequency (percentage) for categorical variables in males and females. A comparison group analysis according to the quartile of DII score was performed for the categorical variables using the Student's *t-test*, ANOVA, or χ^2^ *tests*. A generalized linear mixed model (GLMM) was performed to observe the longitudinal effects of DII and depressive symptoms. To investigate the effects of elevated dietary inflammatory potential, DII categories (with the lowest tertile as the reference) were included in GLMM models to compare high vs. low DII scores on depressive symptoms. Generalized linear mixed models were selected to account for within-subject correlation while allowing subject-specific trajectories. Models included random intercepts for participants. The main analyses were adjusted for age, sex, marital status, dietary intake, smoking status, alcohol use, and physical activity levels, hypertension, diabetes and hypothyroidism. Subgroup analyses were stratified by sex to assess potential moderating effects. All significance levels were set at *p* < 0.05. A sensitivity analysis was conducted to assess the effects of missing values. All analyses were performed using R (version 4.3.2). Missing values were imputed using multiple imputation by chained equations (MICE; “mice” package), employing the predictive mean matching method with 50 imputations and a maximum of 50 iterations. Missing data were primarily observed in the outcome variable GDS score, about 22.2% (*n* = 3,539/15,940 observations). Convergence was confirmed by trace plots of imputed GDS score means and standard deviations, which stabilized within 50 iterations across all 50 imputations with no evidence of trends or drift through iterations.

## Results

**Figure 1:**
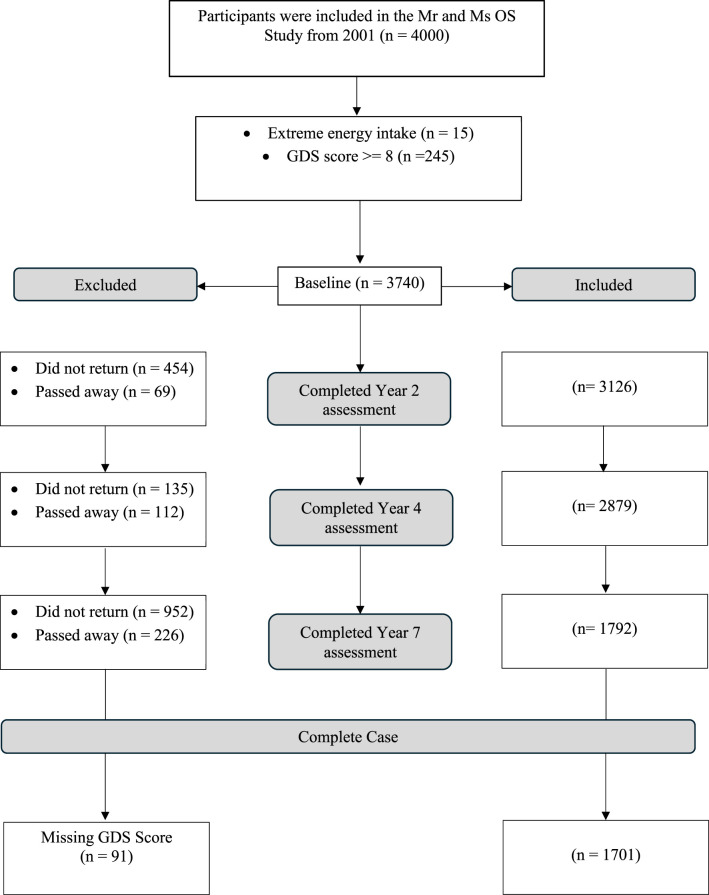
CONSORT-style participants flow chart of recruitment, retention and exclusion.

**Figure 2:**
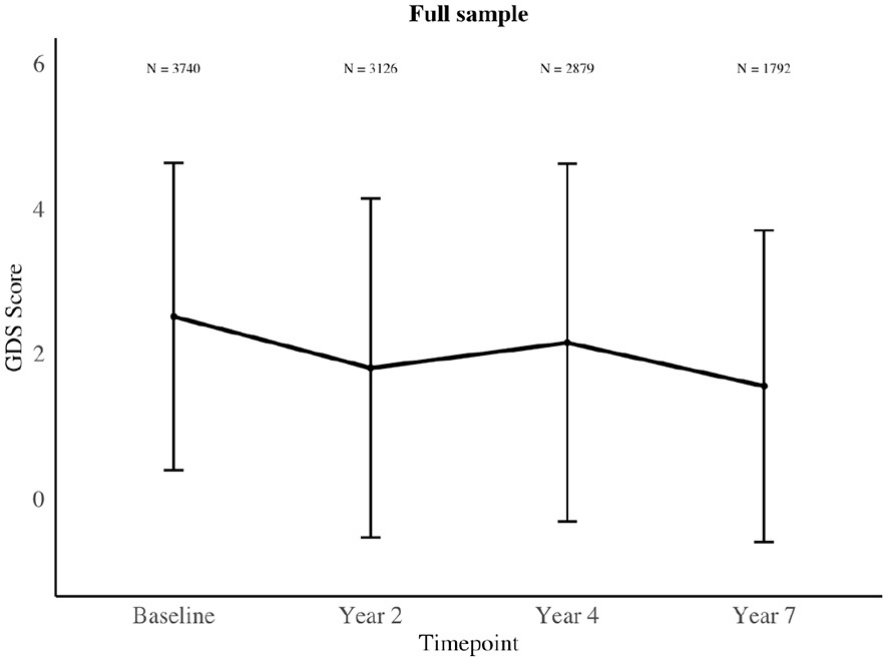
Temporal change in the geriatric depressive scale.

**Figure 3:**
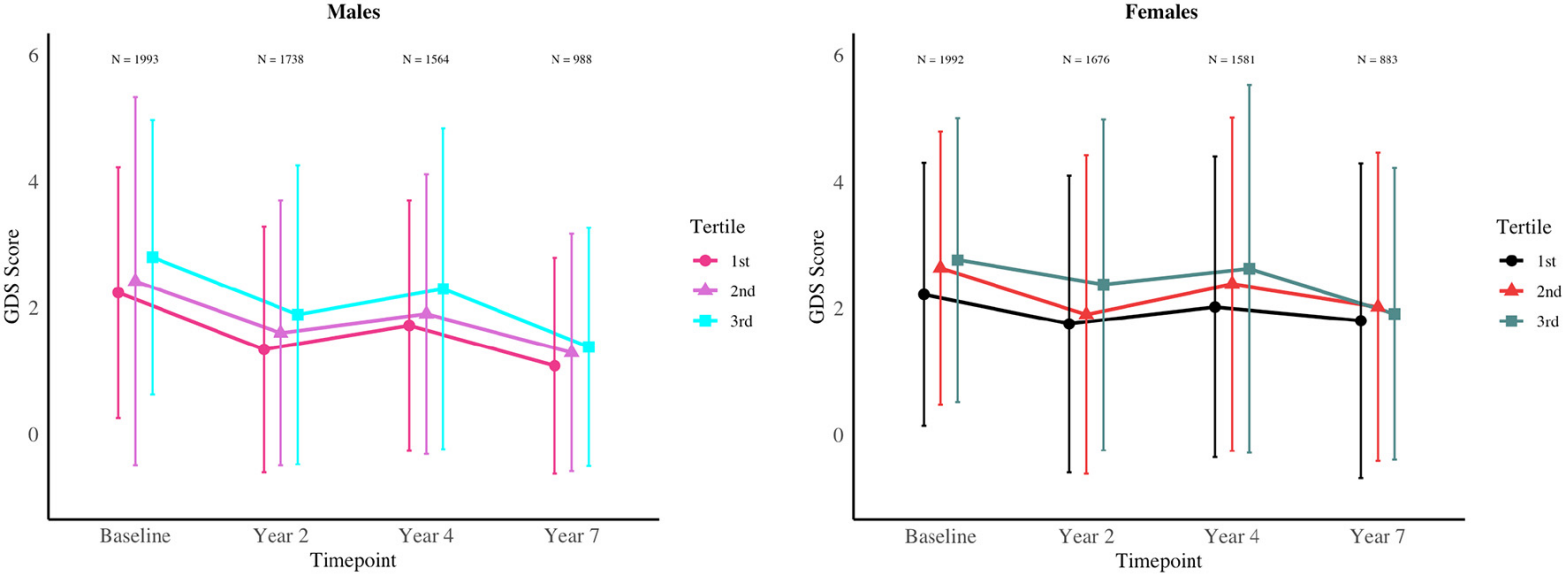
Temporal change in geriatric depressive scale between sexes across dietary inflammatory index by tertile.

Among the 4,000 subjects recruited for Mr and Ms OS study, after excluding individuals with extreme energy intake at baseline (n =5) and those with a GDS score of 8 or above, 3,740 subjects (1,877 males and 1,863 females) completed baseline dietary and depressive symptoms measurements and were followed up for 7 years (Figure 1). The mean (SD) age of males and females was 72.39 (5.01) and 72.58 (5.36), respectively. The mean DII score in males was −0.80 (1.38), lower than in females at −0.14 (1.51). The mean E-DII score in males was −0.03 (0.72), and in females, 0.07 (1.11). The characteristics and depressive symptom measurements across the DII categories in tertiles are presented in Table 1. Over the 7 years, both males and females showed a decrease in depressive symptoms (Figures 2, 3). Participants with a more pro-inflammatory diet had lower physical activity levels (*p* = 0.001) in both sexes, while males, with a mean PASE score of 97.76 (50.42), had higher scores than females, with 85.72 (32.79) (*p* < 0.001).

**Table 1:** Baseline characteristics and depressive symptoms measurement by tertile.

Baseline variables	Total *n* = 3,740	First tertile *n* = 1,233	Second tertile *n* = 1,234	Third tertile *n* = 1,273	*p*
DII, mean (*SD*)	−0.50 (1.47)	−1.78 (0.88)	−0.60 (0.85)	0.83 (1.22)	< 0.001
Age (years), mean (*SD*)	72.41 (5.15)	72.02 (5.10)	72.27 (4.86)	72.92 (5.43)	< 0.001
Sex (F), *n* (%)	1,863 (49.8)	661 (53.6)	568 (46.0)	633 (49.8)	0.001
Married, *n* (%)	2,673 (71.5)	904 (73.3)	909 (73.7)	860 (67.6)	0.001
Current smoker, *n* (%)	243 (6.5)	57 (4.6)	81 (6.6)	105 (8.3)	0.001
Alcohol use, *n* (%)	495 (13.2)	205 (16.6)	166 (13.5)	124 (9.8)	< 0.001
BMI (kg/m^2^), mean (*SD*)	23.69 (3.29)	23.90 (3.27)	23.68 (3.34)	23.51 (3.25)	0.011
PASE score, mean (S*D*)	91.77 (42.98)	94.44 (43.09)	92.88 (44.19)	88.04 (41.43)	0.001
Energy intake (kcal/day), mean (*SD*)	1,844.95 (579.24)	1,835.23 (492.70)	1,854.47 (586.18)	1,844.96 (646.66)	0.712
Protein/day (g), mean (*SD*)	76.64 (33.02)	81.30 (29.39)	77.52 (33.72)	71.25 (34.86)	< 0.001
Carbohydrate/day (g), mean (*SD*)	257.45 (84.02)	257.31 (77.23)	256.55 (84.74)	258.36 (89.51)	0.863
Fat/day (g), mean (*SD*)	58.22 (23.55)	56.04 (18.03)	59.23 (22.76)	59.38 (28.40)	< 0.001
Presence of hypertension, *n* (%)	1,586 (42.4)	25 (2.0)	22 (1.8)	14 (1.1)	0.203
Presence of diabetes, *n* (%)	533 (14.3)	195 (15.8)	164 (13.3)	174 (13.7)	0.154
Presence of hypothyroidism, *n* (%)	61 (1.6)	25 (2.0)	22 (1.8)	14 (1.1)	0.164

p-value represents the results from ANOVA comparison between dietary inflammatory index, dietary inflammatory index (DII) ranges from a negative value to a positive value, where the higher the value, the greater the inflammatory potential.

No significant differences in age were found between the two sexes across all DII scores (*p* = 0.514).

Time was negatively associated with the GDS score [β = −0.125 (0.007), 95% *CI*: −0.139, −0.111]. The DII score was positively associated with the GDS score after adjusting for age, sex, lifestyle factors, and chronic diseases. A one-unit increase in DII was associated with a 0.23-point increase in GDS scores [β = 0.228 (0.028), 95% *CI*: 0.171, 0.285] at year 7. In examining the differences between DII score tertiles (Table 2), the results showed that an increase in the DII score is positively associated with the GDS score, with the second tertile having a significantly higher GDS score [β = 0.182 (0.084), 95% *CI*: 0. 017, 0. 347], and the third tertile showed a further increase in the significant association [β = 0.595 (0.083), 95% *CI*: 0.431, 0.758]. Given that the GDS ranges from 0 to 15 and a 5-point difference distinguishes normal from mild depression, this represents a modest but clinically relevant effect. Convergence diagnostic plots were generated to assess the quality of the imputations (Appendix 1).

**Table 2:** Generalized linear mixed-models of depressive symptoms regressed on DII, time-in-study between tertiles.

Sample	Fixed effects (SE)		First tertile	Second tertile	Third tertile
	DII (β)	95% *CI*			
Unweighted	0.228^**^	(0.171, 0.285)	Ref.	0.182^*^	0.595^**^
IPW weighted	0.128^**^	(0.035, 0.222)			
Male					
Unweighted	0.199^**^	(0.122, 0.277)	Ref.	0.098	0.459^**^
IPW weighted	0.082	(−0.034, 0.198)			
Female					
Unweighted	0.266^**^	(0.184, 0.348)	Ref.	0.229	0.750^**^
IPW weighted	0.170^**^	(0.020, 0.320)			

Fixed effects estimate the influence of DII on depressive symptoms. Models include DII as a continuous variable (column 2) OR as categorical tertiles (columns 3–5), not both simultaneously. The first tertile (lowest inflammatory diet) serves as the reference category; the first tertile indicates the least inflammatory diet, and the third tertile indicates the most inflammatory diet. Analysis was adjusted for age and sex, lifestyle covariates at baseline, and diagnoses of diabetes, hypertension, and hypothyroidism. Significant level (*p* < 0.05) indicated by ^*^, (*p* < 0.001) indicated by ^**^ stabilized inverse probability weights were derived from logistic regression models predicting continued participation at each wave as a function of sociodemographic and health-related characteristics, including age, sex, physical activity, diabetes status, hypertension, body mass index, hypothyroidism, marital status, smoking, and alcohol consumption.

The association between DII and GDS scores was statistically significant across both sexes, with males exhibiting a smaller effect size than females (Table 2). Specifically, a one-unit increase in DII was associated with a 0.2-point rise in GDS scores in males [β = 0.199 (0.039), 95% *CI*: 0.122, 0.277], compared to a 0.27-point increase in females [β = 0.266 (0.042), 95% *CI*: 0.184, 0.348]. When examining the differences in DII scores by tertile across sexes, the results indicated that an increase in DII score is positively associated with GDS scores in the highest tertile, amplifying the significant association in males [β = 0.459 (0.117), 95% *CI*: 0.230, 0.690] and females [β = 0.750 (0.126), 95% *CI*: 0.505, 0.998]. Baseline characteristics by sex and tertile are detailed in Appendix 2.

### Sensitivity analysis

A total of 1,701 subjects (912 males and 789 females) were included for the sensitivity analysis. The mean (SD) age of males and females was 70.92 (4.24) and 70.59 (4.27), respectively. A significant difference between males and females who did not return to the study was observed in that a greater number of males have died (*n* = 309) when compared to females (*n* = 139) (*p* < 0.001). Furthermore, more females reported a loss to follow-up than males (*p* < 0.001), 975 vs. 702, respectively. Males with a more pro-inflammatory diet had higher fat and carbohydrate intake than females, who had lower intake of fat, protein, and carbohydrates. A greater proportion of males were diagnosed with hypertension than those with a more pro-inflammatory diet. In contrast, more females with the least inflammatory diet were diagnosed with hypertension and diabetes. Baseline characteristics are shown in Appendix 3 and, by sex and tertile, in Appendix 4. Time was negatively associated with the GDS score [β = −0.079 (0.008), 95% *CI*: −0.094, −0.064]. The fixed effects of the DII score were positively associated with the GDS score [β = 0.168 (0.025), 95% *CI*: 0.120, 0.216] at year 7. Indicating that a one-unit increase in DII was associated with a 0.17-point increase in GDS scores.

In examining the differences between the DII score by tertile, the results showed the increase in the DII score is positively associated with the GDS score in that the third tertile had a significantly higher GDS score [β = 0.478 (0.073), 95% *CI*: 0.335, 0.622] than the second tertile [β = 0.232 (0.073), 95% *CI*: 0.090, 0.375]. The results of the generalized linear mixed model of the sensitivity analysis were presented in Table 3. Subgroup analyses in males and females revealed that the association between DII and GDS scores was statistically significant after adjustment for age, lifestyle factors, and chronic diseases in both groups. Specifically, a one-unit increase in DII was associated with a 0.16-point rise in GDS scores in males [β = 0.155 (0.042), 95% *CI*: 0.090, 0.219], compared to a 0.19-point increase in females [β = 0.188 (0.036), 95% *CI*: 0.117, 0.259]. In examining differences in DII scores by tertile across sexes, the results showed that the increase in DII score is positively associated with the GDS score in the highest tertile, strengthening the significant association in males [β = 0.459 (0.082), 95% *CI*: 0.269, 0.648]. In females, the increase in dietary inflammatory index was positively associated with GDS score in both the second and third tertile [β = 0.337 (0.113), 95% *CI*: 0.116, 0.557] and [β = 0.525 (0.110), 95% *CI*: 0.309, 0.740], respectively.

**Table 3:** Sensitivity analysis using the generalized linear mixed models of depressive symptoms regressed on DII, time-in-study, between tertiles.

Sample	Fixed effects (SE)		First tertile	Second tertile	Third tertile
	DII (β)	95% *CI*			
Full sample	0.168^**^	(0.120, 0.216)	Ref.c	0.232^**^	0.478^**^
By sex					
Male	0.155^**^	(0.090, 0.219)	Ref.	0.162	0.459^**^
Female	0.188^**^	(0.117, 0.259)	Ref.	0.337^*^	0.525^**^

Fixed effects estimate the influence of DII on depressive symptoms. Models include DII as a continuous variable (column 2) OR as categorical tertiles (columns 3–5), not both simultaneously. The first tertile (lowest inflammatory diet) serves as the reference category; the first tertile indicates the least inflammatory diet, and the third tertile indicates the most inflammatory diet. Analysis was adjusted for age and sex, lifestyle covariates at baseline, and diagnoses of diabetes, hypertension, and hypothyroidism. Significant level (*p* < 0.05) indicated by ^*^, (*p* < 0.001) indicated by ^**^.

Our analysis showed that a significant and consistent association between DII and depressive symptoms persisted over time. To assess potential attrition bias, we employed stabilized inverse probability weighting (IPW) to adjust for attrition, yielding results similar to those of the primary mixed-effects model (Table 2). In the IPW-weighted analysis, higher DII was linked to higher depressive symptom scores (β = 0.229 (0.029), 95% *CI*: 0.173–0.286), with an effective sample size of 97%. This indicated that the observed relationship was minimally influenced by loss to follow-up. Sensitivity analyses applying additive shifts to depressive scores of dropouts (n = 2,208) showed the exposure coefficient changed modestly (β = 0.217–0.242) and remained significant across a range of shifts (δ = −0.5 to +0.5; Appendix 5). These small changes in score variability relative to that observed under the missing-at-random assumption indicate that only large, systematic deviations from it could nullify the association.

## Discussion

This is the first study to examine levels of dietary inflammatory potential and their association with depressive symptoms in the Hong Kong Chinese elderly cohort. Interestingly, despite prior evidence indicating that changes in health conditions (e.g., increased chronic diseases) and social factors (e.g., loneliness and social deprivation) contribute to a higher risk of depression (24–26), a decreasing trend in GDS scores was observed in both males and females over 7 years. A possible explanation is that individuals develop better coping strategies and adapt as they age. Research has shown that skills such as problem-solving and mastery are linked to lower rates of depression (27). Furthermore, previous studies have suggested that depressive symptoms may decrease significantly after menopause in females, particularly in those without a history of depression (28). Another explanation could be attrition, since earlier research has found that depressed individuals are more likely to drop out (29). To evaluate whether the association between DII and depressive symptoms varied over time, an interaction term between DII and follow-up wave was examined. The association remained positive across all time points with statistically significant effects at baseline, Year 2, and Year 4. Although the magnitude of the association appeared attenuated by Year 7, confidence intervals overlapped across waves, and there was no evidence of a reversal in direction. These findings suggest that the DII–depression association was generally stable over time (Appendix 6).

Furthermore, the average DII scores were notably lower than those reported in previous research within Western cultures (30), implying that cultural differences may affect habitual diets and, consequently, dietary inflammation. For instance, the “Western diet,” characterized by high intake of red/processed meats, sugar-sweetened beverages, and refined grains, and low consumption of fruit and vegetables (31), is associated with low-grade inflammation compared with other traditional, healthier dietary patterns (32). It has been shown that culture and traditions can influence food choices (33, 34). For example, rapeseed oil, which contains anti-inflammatory nutrients (35), is most commonly used in the Chinese diet (36), whereas animal fats are more typical in Western diets. Additionally, despite regional differences, the traditional Chinese diet primarily consists of foods with lower inflammatory potential (37). For instance, soy and its derivatives are staple foods in this diet and are known for their high levels of anti-inflammatory isoflavones (38). Wholegrain cereals, recognized for their anti-inflammatory properties (39), also play a significant role in traditional Chinese diets (40). Moreover, foods like seaweed, which have demonstrated anti-inflammatory benefits (41, 42), are commonly used in Chinese cuisine. Therefore, following a more traditional Chinese diet may have contributed to the lower inflammatory index score. However, since many diet-related factors, such as cooking methods and food availability, may influence food choices, future research should employ principal component analysis to better understand the cultural importance of food components and how these factors may impact dietary inflammation and its association with depressive symptoms.

Our results from the Mr and Ms OS survey showed that the DII score was positively linked to depressive symptoms. Additionally, subgroup analyses indicated that the effects of dietary inflammation were further influenced by sex. Specifically, it was observed that the DII score was positively connected to depressive symptoms in both males and females. Our findings aligned with the SUN (Seguimiento Universidad de Navarra) project on middle-aged males and females, which showed that higher DII scores increased the risk of depression by 47% when comparing the most pro-inflammatory diet with the most anti-inflammatory diet (43). The highest tertile of DII score, representing a diet with the greatest pro-inflammatory potential, was associated with higher long-term depressive symptom scores, demonstrating that dietary inflammatory potential is positively related to depressive symptoms regardless of cultural influences.

Regarding sex-specific effects, females had a higher mean DII score than males across all tertiles, suggesting sex differences in dietary behaviors and preferences. For instance, males were found to have stronger preferences for red and processed meat, while females were more likely to consume more vegetables and whole grains and were more susceptible to sweet flavors (44). Consistent with the results reported by the Australian Longitudinal Study on Women's Health and the Whitehall Study II. Respectively, the Australian study reported that an anti-inflammatory diet reduces the risk of depressive symptoms development (30). However, in contrast with the Whitehall II study, which found that a pro-inflammatory diet increased the risk of depressive symptoms among females but not in males (45), our results showed a positive association between dietary inflammatory potential and depressive symptoms in both sexes. While it is well-established that older females have a greater likelihood of depression compared to older males (46), a comparable observation was not detected in our study population.

Furthermore, our results showed that females could be more susceptible to depressive symptoms influenced by diet. For instance, we found that in females, both the second and third tertiles of the DII score were positively associated with longitudinal depressive symptoms. In contrast, only the third tertile of the DII score was associated with longitudinal depressive symptoms in males. Additionally, a stronger association between females with the highest DII score and depressive symptoms when compared to those with lower DII scores. Previous studies have suggested that high-glycemic-index food intake and overconsumption of dietary fat are associated with low-grade systemic inflammation (47). The high levels of simple carbohydrate and fat consumption may elevate dietary inflammatory potential (48, 49), subsequently potentially worsening depressive symptoms.

Notably, males and females have different nutritional profiles. For instance, males in the highest tertile of DII score had the highest fat and carbohydrate consumption, whereas females showed the reverse pattern. Moreover, while males had higher overall food consumption than females, females had a higher overall inflammatory score across all tertiles. It is well documented that males have higher energy expenditure and resting metabolic rates (50), leading to greater food consumption. Despite that, our results showed that females' dietary patterns were more significantly associated with depressive symptoms compared to males, suggesting that although macronutrients may play a critical role in inflammation, there may be potential differences in mechanisms leading to the associations with depressive symptoms. Previous studies in animal models have demonstrated that high-fat and refined carbohydrate consumption may impair neurogenesis and reduce brain-derived neurotrophic factor levels in the hippocampus (51), and modify neurotransmitter release (52), thereby potentially contributing to depression (53). In contrast, a low-carbohydrate diet was associated with reduced risk of depression (54). Nevertheless, our results are limited in their ability to infer the mechanisms underlying the associations. Further studies are warranted to understand the nutritional differences and the underlying mechanisms leading to the sex difference.

Some studies have suggested the sex difference may be due to oxidative stress and inflammatory management. For instance, hormonal influences such as the level of estrogen between sexes and throughout life, like menopause, may influence inflammatory responses and energy balance (55–57), potentially leading to increased susceptibility to depression (58). Another biological difference noted in recent literature involves variations in microbiota composition between males and females (59, 60). Notably, research indicates that gut microbiota may significantly influence brain function and behavior through the microbiota-gut-brain axis. Pro-inflammatory diets could alter microbiota composition (61) and increase the abundance of inflammatory bacteria, such as *Actinobacteria*, in the gut, which has been associated with neuroinflammation (62, 63). For instance, distinct *Actinobacteria phylotypes* have been observed in females with major depression compared to males (60). These findings in hormonal balance and gut microbiota composition may reflect the observed results and warrant further research.

### Implications

This study provides further evidence supporting the role of chronic inflammation as a potential mechanism in the relationship between diets and depressive symptoms. Our findings highlighted that the importance of diet for mental well-being extends beyond dietary patterns and nutrient intake to diet quality. This suggests that dietary inflammation is a modifiable risk factor that should be carefully considered when investigating depression alongside lifestyle and psychosocial factors. The sex-specific results highlighted that when investigating depression, differences in DII score and the potential dietary behaviors could be significant, where sex-specific dietary recommendations for altering dietary inflammation, such as a greater reduction in inflammatory foods in females, should be considered for depression management and prevention.

### Limitations

Several limitations of the current study need to be acknowledged. Firstly, dietary patterns of individuals were collected only at baseline using a food frequency questionnaire, which assumes that participants' nutritional patterns remain stable over the 7-year follow-up period. The lack of repeated measures precludes capturing temporal changes. Non-differential measurement error may lead to regression dilution bias, attenuating the observed association between an exposure and outcome toward the null. Furthermore, this may prevent assessment of within-individual changes in dietary inflammatory potential and introduce exposure misclassification. Consequently, true associations between dietary inflammatory potential and depression may attenuate or bias the observation, particularly if dietary patterns became more (or less) inflammatory over time, but such changes were not reflected in the model. Hence, our observations may not fully reflect the causal relationship and warrant cautious interpretation. However, previous studies have suggested that dietary behaviors established earlier in life remain relatively consistent in the long term, even into very late life, among the Chinese population (64–66). Future studies should aim to include repeated dietary assessment to better capture changes over time and strengthen causal inferences.

Secondly, while the current study utilized the GDS to identify depression at baseline, several factors may have contributed to the declining trend of depressive symptoms observed over 7 years. For instance, previous studies have reported that repeated testing can lead to practice effects [84], and a Chinese study also observed a decreasing trend in depressive symptoms, potentially due to a cohort effect [85]. Thus, the lack of a clinical diagnosis may limit clinical implications and therapeutic potential. Thirdly, some social factors, such as socioeconomic status, education level, and social support, were not measured in the current study. Lower levels in these factors are known to influence depressive symptoms negatively (67) and diet quality (68). The lack of these variables may confound our results and potentially lead to underestimates of the actual effects of dietary inflammation. While Mr and Ms Os' study primarily focuses on the clinical variables related to osteoporosis, this may have led to the unintentional oversight of broader socioeconomic factors. The conceptual model of the analysis is shown in Appendix 7. Future studies should consider including these factors when investigating the association between diet and depressive symptoms to improve understanding of this association. Next, it is essential to acknowledge the loss of follow-up and the potential attrition bias. It was observed that individuals who dropped out of the study reported poorer overall sociodemographic status, higher DII and GDS scores (Appendix 8, 9) and greater depressive symptoms (Appendix 10). This selective loss of more vulnerable individuals likely shifts the remaining analytic sample toward healthier, more resilient survivors, diluting the observable strength of associations between dietary patterns and depression over time. However, inverse probability weighting and sensitivity analyses that account for differential attrition suggested that the relationship between DII and depressive symptoms was robust against potential survival-related bias, reducing concerns about selective dropout. Nevertheless, these attrition patterns may have led to an underestimation of the association between DII score and depressive symptoms. Therefore, the results may not generalize to elderly adults with more severe depressive symptoms or higher dietary inflammation, as these individuals were more likely to drop out.

### Conclusion

In conclusion, despite the limitations of the causal inference, particularly the single baseline dietary assessment, the current study suggests that dietary inflammatory potential is positively associated with depressive symptoms. Moreover, sex may act as a moderator, with females potentially being more affected by inflammatory diets. Adopting a diet with lower inflammatory potential may benefit both mental and physical health outcomes. Public health practitioners should aim to promote the importance of diet quality and an anti-inflammatory diet. Reducing foods with high inflammatory potential is recommended to improve emotional health and reduce the burden of depression in females. Future studies should validate the causal relationship by introducing repeated dietary measures and exploring the underlying mechanisms of sex differences.

## Data Availability

The original contributions presented in the study are included in the article/Supplementary material, further inquiries can be directed to the corresponding author/s.

## References

[1] Custodero C Mankowski RT Lee SA Chen Z Wu S Manini TM . Evidence-based nutritional and pharmacological interventions targeting chronic low-grade inflammation in middle-age and older adults: a systematic review and meta-analysis. Ageing Res Rev. (2018) 46:42–59. doi: 10.1016/j.arr.2018.05.004 29803716 PMC6235673

[2] Shivappa N Steck SE Hurley TG Hussey JR Hebert JR. Designing and developing a literature-derived, population-based dietary inflammatory index. Public Health Nutr. (2014) 17:1689–96. doi: 10.1017/S1368980013002115 23941862 PMC3925198

[3] Shakya PR Melaku YA Shivappa N Hebert JR Adams RJ Page AJ . Dietary inflammatory index (DII(R)) and the risk of depression symptoms in adults. Clin Nutr. (2021) 40:3631–42. doi: 10.1016/j.clnu.2020.12.031 33485704

[4] Li X Chen M Yao Z Zhang T Li Z. Dietary inflammatory potential and the incidence of depression and anxiety: a meta-analysis. J Health Popul Nutr. (2022) 41:24. doi: 10.1186/s41043-022-00303-z 35643518 PMC9148520

[5] Bennett G Bardon LA Gibney ER. A comparison of dietary patterns and factors influencing food choice among ethnic groups living in one locality: a systematic review. Nutrients. (2022) 14:941. doi: 10.3390/nu14050941 35267916 PMC8912306

[6] Li R Zhan W Huang X Zhang Z Zhou M Bao W . Association of dietary inflammatory index (DII) and depression in the elderly over 55 years in Northern China: analysis of data from a multicentre, cohort study. BMJ Open. (2022) 12:e056019. doi: 10.1136/bmjopen-2021-056019 PMC9024263 35450904

[7] Adjibade M Andreeva VA Lemogne C Touvier M Shivappa N Hebert JR . The inflammatory potential of the diet is associated with depressive symptoms in different subgroups of the general population. J Nutr. (2017) 147:879–87. doi: 10.3945/jn.116.245167 28356432 PMC6636662

[8] Phillips CM Shivappa N Hebert JR Perry IJ. Dietary inflammatory index and mental health: a cross-sectional analysis of the relationship with depressive symptoms, anxiety and well-being in adults. Clin Nutr. (2018) 37:1485–91. doi: 10.1016/j.clnu.2017.08.029 28912008

[9] Shivappa N Godos J Hebert JR Wirth MD Piuri G Speciani AF . Dietary inflammatory index and cardiovascular risk and mortality-a meta-analysis. Nutrients. (2018) 10:200. doi: 10.3390/nu10020200 29439509 PMC5852776

[10] Lee H-cB Chiu HFK Kwow WY Leung CM. Chinese elderly and the GDS short form: a preliminary study. J Aging Ment Health. (1993) 14:37–42.

[11] Liu B Woo J Tang N Ng K Ip R Yu A. Assessment of total energy expenditure in a Chinese population by a physical activity questionnaire: examination of validity. Int J Food Sci Nutr. (2001) 52:269–82. doi: 10.1080/09637480120044138 11400476

[12] Chan RSM Yu BWM Leung J Lee JSW Auyeung TW Kwok T . How dietary patterns are related to inflammaging and mortality in community-dwelling older Chinese adults in Hong Kong—a prospective analysis. J Nutr Health Aging. (2019) 23:181–94. doi: 10.1007/s12603-018-1143-0 30697629 PMC12280441

[13] Yang Y Wang Y Pan X. China Food Composition Table. Beijing: Peking University (2009).

[14] Safety CfF. Nutrient Information Inquiry Hong Kong: HKSAR. Available online at: https://www.cfs.gov.hk/english/nutrient/presearch3.php (Accessed May 01, 2026).

[15] Administration TFaD. Food Composition Database for Consumer (Update 1 v1): Taiwan Food and Drug Administration (2023). Available online at: https://consumer.fda.gov.tw/Food/TFND.aspx?nodeID=178 (Accessed May 01, 2026).

[16] Raw P. Composition of Foods Raw, Processed, Prepared USDA National Nutrient Database for Standard Reference, Release 25. United States Department of Agriculture (USDA) (2012).

[17] Shivappa N Steck SE Hurley TG Hussey JR Ma Y Ockene IS . A population-based dietary inflammatory index predicts levels of C-reactive protein in the seasonal variation of blood cholesterol study (SEASONS). Public Health Nutr. (2014) 17:1825–33. doi: 10.1017/S1368980013002565 24107546 PMC3983179

[18] Willett WC Howe GR Kushi LH. Adjustment for total energy intake in epidemiologic studies. Am J Clin Nutr. (1997) 65(4 Suppl):1220S-8S. doi: 10.1093/ajcn/65.4.1220S 9094926

[19] Mitchell AJ Bird V Rizzo M Meader N. Diagnostic validity and added value of the geriatric depression scale for depression in primary care: a meta-analysis of GDS30 and GDS15. J Affect Disord. (2010) 125:10–7. doi: 10.1016/j.jad.2009.08.019 19800132

[20] Huang CQ Dong BR Lu ZC Yue JR Liu QX. Chronic diseases and risk for depression in old age: a meta-analysis of published literature. Ageing Res Rev. (2010) 9:131–41. doi: 10.1016/j.arr.2009.05.005 19524072

[21] Glaesmer H Riedel-Heller S Braehler E Spangenberg L Luppa M. Age- and gender-specific prevalence and risk factors for depressive symptoms in the elderly: a population-based study. Int Psychogeriatr. (2011) 23:1294–300. doi: 10.1017/S1041610211000780 21729425

[22] Nuguru SP Rachakonda S Sripathi S Khan MI Patel N Meda RT. Hypothyroidism and depression: a narrative review. Cureus. (2022) 14:e28201. doi: 10.7759/cureus.28201 36003348 PMC9392461

[23] Washburn RA Smith KW Jette AM Janney CA. The physical activity scale for the elderly (PASE): development and evaluation. J Clin Epidemiol. (1993) 46:153–62. doi: 10.1016/0895-4356(93)90053-4 8437031

[24] Zenebe Y Akele B W/Selassie M Necho M. Prevalence and determinants of depression among old age: a systematic review and meta-analysis. Ann Gen Psychiatry. (2021) 20:55. doi: 10.1186/s12991-021-00375-x 34922595 PMC8684627

[25] Zhu Y Li C Xie W Zhong B Wu Y Blumenthal JA. Trajectories of depressive symptoms and subsequent cognitive decline in older adults: a pooled analysis of two longitudinal cohorts. Age Ageing. (2022) 51:afab191. doi: 10.1093/ageing/afab191 34657957

[26] Paiva TC Soares L Faria AL. Depression in elderly people. Encyclopedia. (2023) 3:677–86. doi: 10.3390/encyclopedia3020048

[27] Jeon HS Dunkle RE. Stress and depression among the oldest-old: a longitudinal analysis. Res Aging. (2009) 31:661–87. doi: 10.1177/0164027509343541 21572921 PMC3092309

[28] Freeman EW. Depression in the menopause transition: risks in the changing hormone milieu as observed in the general population. Womens Midlife Health. (2015) 1:2. doi: 10.1186/s40695-015-0002-y 30766689 PMC6214217

[29] Proudfoot J Clarke J Birch M-R Whitton AE Parker G Manicavasagar V . Impact of a mobile phone and web program on symptom and functional outcomes for people with mild-to-moderate depression, anxiety and stress: a randomised controlled trial. BMC Psychiatry. (2013) 13:312. doi: 10.1186/1471-244X-13-312 24237617 PMC4225666

[30] Shivappa N Schoenaker DA Hebert JR Mishra GD. Association between inflammatory potential of diet and risk of depression in middle-aged women: the Australian Longitudinal Study on Women's Health. Br J Nutr. (2016) 116:1077–86. doi: 10.1017/S0007114516002853 27498949

[31] Zhang H Li M Mo L Luo J Shen Q Quan W. Association between western dietary patterns, typical food groups, and behavioral health disorders: an updated systematic review and meta-analysis of observational studies. Nutrients. (2023) 16:125. doi: 10.3390/nu16010125 38201955 PMC10780533

[32] Norde MM Collese TS Giovannucci E Rogero MM. A posteriori dietary patterns and their association with systemic low-grade inflammation in adults: a systematic review and meta-analysis. Nutr Rev. (2021) 79:331–50. doi: 10.1093/nutrit/nuaa010 32417914

[33] Köster EP. Diversity in the determinants of food choice: a psychological perspective. Food Qual Prefer. (2009) 20:70–82. doi: 10.1016/j.foodqual.2007.11.002

[34] Chen PJ Antonelli M. Conceptual models of food choice: influential factors related to foods, individual differences, and society. Foods. (2020) 9:1898. doi: 10.3390/foods9121898 33353240 PMC7766596

[35] Shen J Liu Y Wang X Bai J Lin L Luo F . A comprehensive review of health-benefiting components in rapeseed oil. Nutrients. (2023) 15:999. doi: 10.3390/nu15040999 36839357 PMC9962526

[36] Yang R Xue L Zhang L Wang X Qi X Jiang J . Phytosterol contents of edible oils and their contributions to estimated phytosterol intake in the Chinese diet. Foods. (2019) 8:334. doi: 10.3390/foods8080334 31404986 PMC6723959

[37] Niu J Li B Zhang Q Chen G Papadaki A. Exploring the traditional Chinese diet and its association with health status—a systematic review. Nutr Rev. (2024) 152:1–15. doi: 10.1093/nutrit/nuae013 PMC11723156 38452296

[38] Das D Sarkar S Borsingh Wann S Kalita J Manna P. Current perspectives on the anti-inflammatory potential of fermented soy foods. Food Res Int. (2022) 152:110922. doi: 10.1016/j.foodres.2021.110922 35181093

[39] Sang S Idehen E Zhao Y Chu Y. Emerging science on whole grain intake and inflammation. Nutr Rev. (2020) 78:21–8. doi: 10.1093/nutrit/nuz079 32728755

[40] Huang L Wang H Wang Z Zhang J Zhang B Ding G. Regional disparities in the association between cereal consumption and metabolic syndrome: results from the China Health and Nutrition Survey. Nutrients. (2019) 11:764. doi: 10.3390/nu11040764 30939825 PMC6521195

[41] Kumar SA Brown L. Seaweeds as potential therapeutic interventions for the metabolic syndrome. Rev Endocr Metab Disord. (2013) 14:299–308. doi: 10.1007/s11154-013-9254-8 23959342

[42] Vaishnudevi D Viswanathan P. Seaweed polysaccharides—new therapeutic insights against the inflammatory response in diabetic nephropathy. Antiinflamm Antiallergy Agents Med Chem. (2017) 15:178–90. doi: 10.2174/1871523016666170217104226 28215166

[43] Sanchez-Villegas A Ruiz-Canela M de la Fuente-Arrillaga C Gea A Shivappa N Hebert JR . Dietary inflammatory index, cardiometabolic conditions and depression in the Seguimiento Universidad de Navarra cohort study. Br J Nutr. (2015) 114:1471–9. doi: 10.1017/S0007114515003074 26344165

[44] Feraco A Armani A Amoah I Guseva E Camajani E Gorini S . Assessing gender differences in food preferences and physical activity: a population-based survey. Front Nutr. (2024) 11:1348456. doi: 10.3389/fnut.2024.1348456 38445208 PMC10912473

[45] Akbaraly T Kerlau C Wyart M Chevallier N Ndiaye L Shivappa N . Dietary inflammatory index and recurrence of depressive symptoms: results from the Whitehall II Study. Clin Psychol Sci. (2016) 4:1125–34. doi: 10.1177/2167702616645777 28070452 PMC5218819

[46] Girgus JS Yang K Ferri CV. The gender difference in depression: are elderly women at greater risk for depression than elderly men? Geriatrics (Basel). (2017) 2:35. doi: 10.3390/geriatrics2040035 31011045 PMC6371140

[47] Galland L. Diet and inflammation. Nutr Clin Pract. (2010) 25:634–40. doi: 10.1177/0884533610385703 21139128

[48] King DE Egan BM Geesey ME. Relation of dietary fat and fiber to elevation of C-reactive protein. Am J Cardiol. (2003) 92:1335–9. doi: 10.1016/j.amjcard.2003.08.020 14636916

[49] Hert KA Fisk PS 2nd Rhee YS Brunt AR. Decreased consumption of sugar-sweetened beverages improved selected biomarkers of chronic disease risk among US adults: 1999 to 2010. Nutr Res. (2014) 34:58–65. doi: 10.1016/j.nutres.2013.10.005 24418247

[50] Arciero PJ Goran MI Poehlman ET. Resting metabolic rate is lower in women than in men. J Appl Physiol. (1985) 75:2514–20. doi: 10.1152/jappl.1993.75.6.2514 8125870

[51] Kanoski SE Davidson TL. Western diet consumption and cognitive impairment: links to hippocampal dysfunction and obesity. Physiol Behav. (2011) 103:59–68. doi: 10.1016/j.physbeh.2010.12.003 21167850 PMC3056912

[52] Jacques A Chaaya N Beecher K Ali SA Belmer A Bartlett S. The impact of sugar consumption on stress driven, emotional and addictive behaviors. Neurosci Biobehav Rev. (2019) 103:178–99. doi: 10.1016/j.neubiorev.2019.05.021 31125634

[53] Yu H Chen ZY. The role of BDNF in depression on the basis of its location in the neural circuitry. Acta Pharmacol Sin. (2011) 32:3–11. doi: 10.1038/aps.2010.184 21131999 PMC4003317

[54] Cheng Z Fu F Lian Y Zhan Z Zhang W. Low-carbohydrate-diet score, dietary macronutrient intake, and depression among adults in the United States. J Affect Disord. (2024) 352:125–32. doi: 10.1016/j.jad.2024.02.054 38367707

[55] Straub RH. The complex role of estrogens in inflammation. Endocr Rev. (2007) 28:521–74. doi: 10.1210/er.2007-0001 17640948

[56] Monteiro R Teixeira D Calhau C. Estrogen signaling in metabolic inflammation. Mediators Inflamm. (2014) 2014:615917. doi: 10.1155/2014/615917 25400333 PMC4226184

[57] Camon C Garratt M Correa SM. Exploring the effects of estrogen deficiency and aging on organismal homeostasis during menopause. Nat Aging. (2024) 4:1731–44. doi: 10.1038/s43587-024-00767-0 39672893 PMC11785355

[58] Alblooshi S Taylor M Gill N. Does menopause elevate the risk for developing depression and anxiety? Results from a systematic review. Australas Psychiatry. (2023) 31:165–73. doi: 10.1177/10398562231165439 36961547 PMC10088347

[59] Niemela L Lamoury G Carroll S Morgia M Yeung A Oh B. Exploring gender differences in the relationship between gut microbiome and depression—a scoping review. Front Psychiatry. (2024) 15:1361145. doi: 10.3389/fpsyt.2024.1361145 38439790 PMC10910028

[60] Chen JJ Zheng P Liu YY Zhong XG Wang HY Guo YJ . Sex differences in gut microbiota in patients with major depressive disorder. Neuropsychiatr Dis Treat. (2018) 14:647–55. doi: 10.2147/NDT.S159322 29520144 PMC5833751

[61] Lin D Peters BA Friedlander C Freiman HJ Goedert JJ Sinha R . Association of dietary fibre intake and gut microbiota in adults. Br J Nutr. (2018) 120:1014–22. doi: 10.1017/S0007114518002465 30355393 PMC8451428

[62] Liu P Liu Z Wang J Wang J Gao M Zhang Y . Immunoregulatory role of the gut microbiota in inflammatory depression. Nat Commun. (2024) 15:3003. doi: 10.1038/s41467-024-47273-w 38589368 PMC11001948

[63] Gao M Wang J Liu P Tu H Zhang R Zhang Y . Gut microbiota composition in depressive disorder: a systematic review, meta-analysis, and meta-regression. Transl Psychiatry. (2023) 13:379. doi: 10.1038/s41398-023-02670-5 38065935 PMC10709466

[64] Lai JS Cheng GH Chong YS Chong MF Koh WP. Longitudinal dietary trajectories with cognitive and psychosocial well-being in Chinese adults aged 85 years and older in Singapore. Innov Aging. (2023) 7:igad036. doi: 10.1093/geroni/igad036 37228450 PMC10205470

[65] Batis C Sotres-Alvarez D Gordon-Larsen P Mendez MA Adair L Popkin B. Longitudinal analysis of dietary patterns in Chinese adults from 1991 to 2009. Br J Nutr. (2014) 111:1441–51. doi: 10.1017/S0007114513003917 24331247 PMC3966951

[66] Xu C Lee YH Jeune S Shelley M. Changing dietary patterns among Chinese older adults: a rural-urban comparative analysis (2008–2018). Int J Behav Med. (2025) 47:1–12. doi: 10.1007/s12529-025-10388-5 40796723

[67] Jespersen A Madden RA Whalley HC Reynolds RM Lawrie SM McIntosh AM . Socioeconomic status and depression—a systematic review. Epidemiol Rev. (2025) 47:mxaf011. doi: 10.1093/epirev/mxaf011 40643383 PMC12448611

[68] Nazri NS Vanoh D Leng SK. Malnutrition, low diet quality and its risk factors among older adults with low socio-economic status: a scoping review. Nutr Res Rev. (2021) 34:107–16. doi: 10.1017/S0954422420000189 32727634

