# Supplementary material for: Dietary inflammatory potential in depressive symptoms in older Chinese people in Hong Kong: a cohort study
Source: Front Nutr. 2026 Jun 1;13:1831232. doi: 10.3389/fnut.2026.1831232 (PMC13265356; doi:10.3389/fnut.2026.1831232)
Supplement: Supplementary file 1 [file Table_1.docx]

*
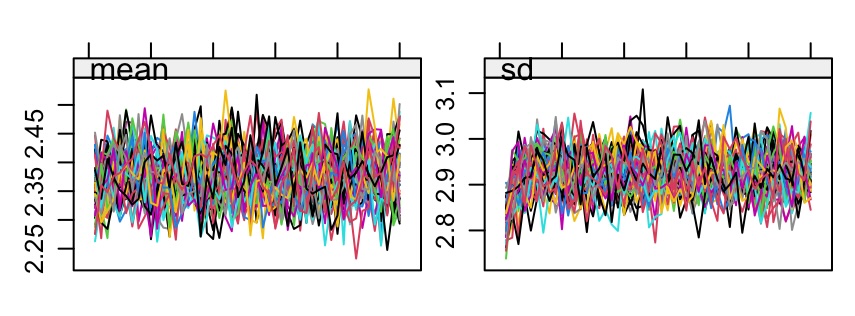
*

Appendix 1. Visual representation of the GDS score mean and standard deviation across iterations.

Appendix 2. Full-sample baseline characteristics and depressive symptom measurements by tertile for males and females.

|  | Total sample | Male | | | Female | | |  |
| --- | --- | --- | --- | --- | --- | --- | --- | --- |
| Baseline variables | n = 3740 | *1^st^ Tertile*  n = 610 | *2^nd^ Tertile*  n = 705 | *3^rd^ Tertile*  n =678 | *1^st^ Tertile*  n= 704 | *2^nd^ Tertile*  n = 610 | *3^rd^ Tertile*  n = 677 | p |
| **DII, Mean (SD)** | -0.50 (1.47) | -2.02 (0.87) | -0.88 (0.86) | 0.39 (1.18) | -1.55 (0.85) | -0.19 (0.69) | 1.36 (1.10) | <0.001 |
| **Age (years), Mean (SD)** | 72.41 (5.15) | 72.03 (4.85) | 72.38 (4.98) | 72.73 (5.16) | 72.06 (5.30) | 72.44 (4.94) | 73.25 (5.72) | <0.001 |
| **Sex (F), n (%)** | 2673 (71.5) | 552 (90.5) | 615 (87.2) | 587 (86.6) | 405 (57.5) | 342 (56.1) | 316 (46.7) | <0.001 |
| **Married, n (%)** | 243 (6.5) | 57 (9.3) | 77 (10.9) | 102 (15.0) | 10 (1.4) | 13 (2.1) | 14 (2.1) | <0.001 |
| **Current smoker, n (%)** | 495 (13.2) | 193 (31.6) | 166 (23.5) | 110 (16.2) | 23 (3.3) | 11 (1.8) | 17 (2.5) | <0.001 |
| **Alcohol Use, n (%)** | 23.69 (3.29) | 23.68 (2.91) | 23.44 (3.26) | 23.24 (3.12) | 24.09 (3.51) | 23.97 (3.40) | 23.71 (3.42) | <0.001 |
| **BMI (kg/m^2^), Mean (SD)** | 91.77 (42.98) | 101.09 (51.03) | 98.61 (50.35) | 92.49 (49.20) | 88.22 (33.73) | 84.82 (33.44) | 82.47 (31.78) | <0.001 |
| **PASE score, Mean (SD)** | 1844.95 (579.24) | 2043.54 (485.08) | 2074.76 (585.30) | 2163.02 (633.48) | 1649.78 (427.76) | 1574.83 (443.88) | 1519.29 (482.67) | <0.001 |
| **Energy intake (Kcal/day), Mean (SD)** | 76.64 (33.02) | 63.34 (18.06) | 67.28 (22.58) | 71.47 (29.24) | 49.51 (15.45) | 49.15 (18.22) | 47.18 (22.63) | <0.001 |
| **Protein/day (g), Mean (SD)** | 257.45 (84.02) | 90.64 (30.72) | 87.19 (35.26) | 84.72 (37.30) | 72.64 (25.74) | 65.38 (27.15) | 57.29 (26.27) | <0.001 |
| **Carbohydrate/day (g), Mean (SD)** | 58.22 (23.55) | 281.53 (79.06) | 282.54 (87.59) | 295.40 (90.74) | 236.01 (69.72) | 222.83 (66.79) | 220.11 (70.31) | <0.001 |
| **Fat/ day (g), Mean (SD)** | 1586 (42.4) | 262 (43.0) | 274 (38.9) | 296 (43.7) | 319 (45.3) | 259 (42.5) | 290 (42.8) | 0.261 |
| **Presence of Hypertension, n (%)** | 533 (14.3) | 104 (17.0) | 103 (14.6) | 85 (12.5) | 111 (15.8) | 70 (11.5) | 103 (15.2) | 0.051 |
| **Presence of Diabetes, n (%)** | 61 (1.6) | 10 (1.6) | 5 (0.7) | 3 (0.4) | 18 (2.6) | 17 (2.8) | 13 (1.9) | 0.002 |

p-value represents the results from t-tests comparing males and females; Dietary Inflammatory Index (DII) ranges from a negative value to a positive value, where the higher the value, the greater the inflammatory potential

Appendix 3. Per-protocol baseline descriptive statistics by tertile

|  | Total  n = 1701 | 1^st^ Tertile  n = 561 | 2^nd^ Tertile  n = 561 | 3^rd^ Tertile  n = 578 | p |
| --- | --- | --- | --- | --- | --- |
| **DII, Mean (SD)** | -0.69 (1.42) | -1.95 (0.86) | -0.78 (0.82) | 0.61 (1.16) | <0.001 |
| **Age (years), Mean (SD)** | 789 (46.4) | 280 (49.9) | 234 (41.7) | 274 (47.4) | 0.019 |
| **Sex (F), n (%)** | 70.77 (4.26) | 70.49 (4.29) | 70.91 (4.17) | 70.93 (4.30) | 0.151 |
| **Married, n (%)** | 1316 (77.4) | 449 (80.0) | 439 (78.3) | 428 (74.0) | 0.046 |
| **Current smoker, n (%)** | 84 (4.9) | 25 (4.5) | 29 (5.2) | 30 (5.2) | 0.811 |
| **Alcohol Use, n (%)** | 242 (14.2) | 103 (18.4) | 79 (14.1) | 60 (10.4) | 0.001 |
| **BMI (kg/m^2^), Mean (SD)** | 23.81 (3.10) | 23.93 (3.10) | 23.84 (3.18) | 23.66 (3.02) | 0.32 |
| **PASE score, Mean (SD)** | 98.60 (44.80) | 101.08 (46.25) | 99.53 (45.14) | 95.34 (42.90) | 0.081 |
| **Energy intake (Kcal/day), Mean (SD)** | 1891.74 (585.26) | 1890.85 (512.28) | 1893.72 (583.37) | 1890.98 (651.25) | 0.996 |
| **Protein/day (g), Mean (SD)** | 79.72 (33.51) | 85.08 (30.99) | 79.81 (32.23) | 74.41 (36.23) | <0.001 |
| **Carbohydrate/day (g), Mean (SD)** | 59.31 (23.61) | 57.64 (18.75) | 60.60 (22.86) | 59.71 (28.08) | 0.098 |
| **Fat/ day (g), Mean (SD)** | 264.07 (85.60) | 264.41 (79.57) | 261.19 (84.96) | 266.53 (91.79) | 0.571 |
| **Presence of Hypertension, n (%)** | 673 (39.6) | 239 (42.6) | 201 (35.8) | 232 (40.1) | 0.063 |
| **Presence of Diabetes, n (%)** | 219 (12.9) | 88 (15.7) | 67 (11.9) | 64 (11.1) | 0.048 |
| **Presence of Hypothyroidism, n (%)** | 27 (1.6) | 10 (1.8) | 10 (1.8) | 7 (1.2) | 0.671 |

*p-value represents the results from ANOVA comparing the tertiles.*

Appendix 4. Per-protocol baseline characteristics and depressive symptom measurement by tertile for males and females.

|  | Total sample | Males | | | Females | | |  |
| --- | --- | --- | --- | --- | --- | --- | --- | --- |
| Baseline variables | n = 1701 | *1^st^ Tertile*  n = 281 | *2^nd^ Tertile*  n = 327 | *3^rd^ Tertile*  n = 304 | *1^st^ Tertile*  n = 280 | *2^nd^ Tertile*  n = 234 | *3^rd^ Tertile*  n = 274 | p |
| **DII, Mean (SD)** | -0.69 (1.42) | -2.16 (0.84) | -1.05 (0.82) | 0.14 (1.07) | -1.74 (0.83) | -0.41 (0.66) | 1.14 (1.01) | <0.001 |
| **Age (years), Mean (SD)** | 70.77 (4.26) | 70.70 (4.29) | 70.87 (4.21) | 71.21 (4.23) | 70.28 (4.29) | 70.96 (4.13) | 70.61 (4.35) | 0.103 |
| **Sex (F), n (%)** | 1316 (77.4) | 268 (95.4) | 296 (90.5) | 274 (90.1) | 181 ( 64.6) | 143 ( 61.1) | 154 ( 56.2) | <0.001 |
| **Married, n (%)** | 84 (4.9) | 21 (7.5) | 25 (7.6) | 27 (8.9) | 4 (1.4) | 4 (1.7) | 3 (1.1) | <0.001 |
| **Current smoker, n (%)** | 242 (14.2) | 92 (32.7) | 76 (23.2) | 53 (17.4) | 11 (3.9) | 3 (1.3) | 7 (2.6) | <0.001 |
| **Alcohol Use, n (%)** | 23.81 (3.10) | 23.66 (2.71) | 23.69 (3.07) | 23.50 (2.84) | 24.20 (3.43) | 24.07 (3.31) | 23.83 (3.20) | 0.005 |
| **BMI (kg/m^2^), Mean (SD)** | 98.60  (44.80) | 109.07 (52.97) | 106.31 (50.16) | 103.60  (50.37) | 93.06  (36.73) | 90.05  (34.98) | 86.18  (30.24) | <0.001 |
| **PASE score, Mean (SD)** | 1891.74  (585.26) | 2080.14 (505.26) | 2093.05 (589.31) | 2205.37 (640.37) | 1700.90 (445.24) | 1615.17 (445.37) | 1542.17 (457.28) | <0.001 |
| **Energy intake (Kcal/day), Mean (SD)** | 79.72 (33.51) | 93.60 (32.27) | 87.88 (34.24) | 88.04 (39.12) | 76.54 (27.14) | 68.54 (25.23) | 59.28 (25.28) | <0.001 |
| **Protein/day (g), Mean (SD)** | 264.07  (85.60) | 286.46 (79.82) | 285.51 (89.19) | 305.60  (93.41) | 242.28 (73.02) | 227.20 (65.03) | 223.19 (67.36) | <0.001 |
| **Carbohydrate/day (g), Mean (SD)** | 59.31 (23.61) | 64.52 (18.46) | 67.93 (22.60) | 70.60 (28.49) | 50.73 (16.37) | 50.35 (19.00) | 47.62 (22.07) | <0.001 |
| **Fat/ day (g), Mean (SD)** | 673 (39.6) | 114 (40.6) | 118 (36.1) | 118 (38.8) | 125 (44.6) | 83 (35.5) | 114 (41.6) | 0.282 |
| **Presence of Hypertension, n (%)** | 219 (12.9) | 47 (16.7) | 44 (13.5) | 27 (8.9) | 41 (14.6) | 23 (9.8) | 37 (13.5) | 0.020 |
| **Presence of Diabetes, n (%)** | 27 (1.6) | 2 (0.7) | 5 (1.5) | 1 (0.3) | 8 (2.9) | 5 (2.1) | 6 (2.2) | 0.50 |

p-value represents the results from t-tests comparing males and females; Dietary Inflammatory Index (DII) ranges from a negative value to a positive value, where the higher the value, the greater the inflammatory potential

Appendix 5. Sensitivity analysis conducted to assess the robustness of the association between the Dietary Inflammatory Index and depressive symptoms under varying hypothetical shifts.

| Shift | Adjusted Estimate |
| --- | --- |
| -0.5 | 0.22645354 |
| -0.4 | 0.22704113 |
| -0.3 | 0.22762965 |
| -0.2 | 0.22821918 |
| -0.1 | 0.22880977 |
| 0 | 0.2294015 |
| 0.1 | 0.22999444 |
| 0.2 | 0.23058865 |
| 0.3 | 0.23118419 |
| 0.4 | 0.23178114 |
| 0.5 | 0.23237957 |

*The original estimate from the Generalized Linear Mixed Model was 0.228, and the sensitivity analysis reveals how different shifts affect this associations*

Appendix 6. Interaction between DII and Time on Depressive symptoms scores

| DII x Time | 𝛽 | SE | 95% CI |
| --- | --- | --- | --- |
| Baseline | **0.176** | 0.044 | (0.09, 0.26) |
| Year 2 | **0.104** | 0.044 | (0.02, 0.19) |
| Year 4 | **0.143** | 0.044 | (0.06, 0.23) |
| Year 7 | 0.059 | 0.044 | (-0.03, 0.15) |


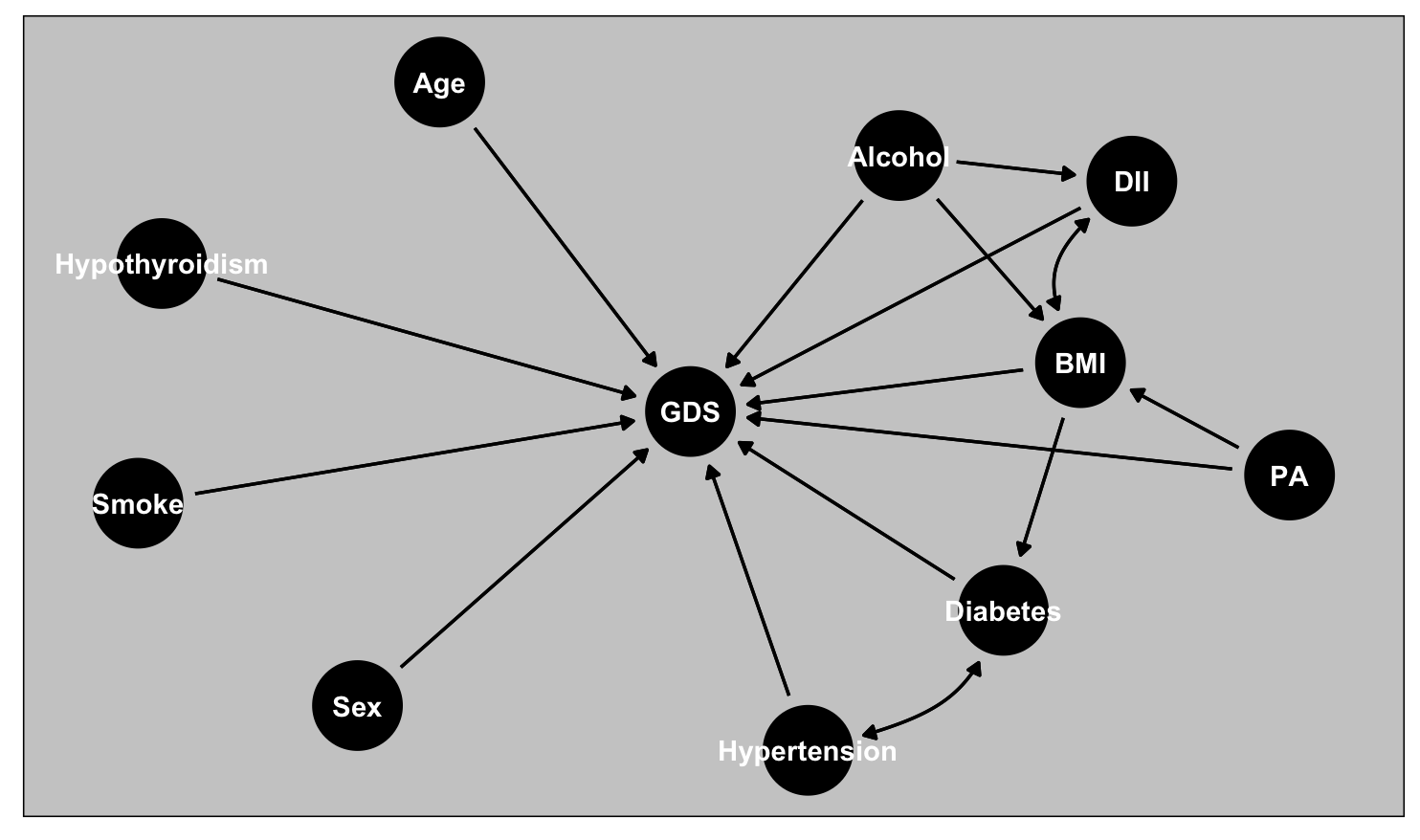


Appendix 7. Directed acyclic graph of the conceptual model of analysis

Appendix 8. Baseline descriptive statistics of individuals were loss of follow-up at 7 years

|  | Returned | Dropped out |  |
| --- | --- | --- | --- |
| n | 1701 | 1948 | p |
| **DII, Mean (SD)** | -0.69 (1.42) | -0.35 (1.47) | <0.001 |
| **Age (years), Mean (SD)** | 789 (46.4) | 1018 (52.3) | <0.001 |
| **Sex (F), n (%)** | 70.77 (4.26) | 73.87 (5.44) | <0.001 |
| **Married, n (%)** | 1316 (77.4) | 1286 (66.0) | <0.001 |
| **Current smoker, n (%)** | 84 (4.9) | 154 (7.9) | <0.001 |
| **Alcohol Use, n (%)** | 242 (14.2) | 243 (12.5) | 0.13 |
| **BMI (kg/m^2^), Mean (SD)** | 23.81 (3.10) | 23.61 (3.45) | 0.059 |
| **PASE score, Mean (SD)** | 98.60 (44.80) | 85.93 (40.77) | <0.001 |
| **Energy intake (Kcal/day), Mean (SD)** | 1891.74 (585.26) | 1803.32 (570.73) | <0.001 |
| **Protein/day (g), Mean (SD)** | 79.72 (33.51) | 73.91 (32.34) | <0.001 |
| **Fat/ day (g), Mean (SD)** | 59.31 (23.61) | 57.26 (23.47) | 0.009 |
| **Carbohydrate/day (g), Mean (SD)** | 264.07 (85.60) | 251.44 (81.99) | <0.001 |
| **Presence of Hypertension, n (%)** | 673 (39.6) | 873 (44.8) | 0.002 |
| **Presence of Diabetes, n (%)** | 219 (12.9) | 305 (15.7) | 0.019 |
| **Presence of Hypothyroidism, n (%)** | 27 (1.6) | 33 (1.7) | 0.903 |
| **GDS Score, Mean (SD)** | 2.25 (1.99) | 2.74 (2.21) | <0.001 |

p-value represents the results from the *t-test comparing individuals who returned for follow-up at 7 years and individuals who dropped out*.

Appendix 9. Dropout rate compared between DII Tertiles

| DII tertile | Dropout Rate | χ² | p |
| --- | --- | --- | --- |
| 1^st^ Tertile | 0.4712084 | 22.617 | < 0.001 |
| 2^nd^ Tertile | 0.5235008 |  |  |
| 3^rd^ Tertile | 0.5660377 |  |  |

Appendix 10. Logistic regression association between loss of follow-up and DII Tertile, Depressive symptoms score

|  |  | 95% CI | |
| --- | --- | --- | --- |
|  | OR | Lower | Upper |
| DII Tertile | **1.17*** | 1.077 | 1.27 |
|  |  |  |  |
| GDS Score | **1.108*** | 1.073 | 1.145 |

Analyses were adjusted by age and sex. * indicates significant level (p <0.05)

Appendix 11. Per-protocol baseline macro- and micronutrient consumption of male and female participants by tertile

|  |  | Male | | | Female | | |  |
| --- | --- | --- | --- | --- | --- | --- | --- | --- |
| **Micronutrients** (Mean(SD)) | Total  n= 1701 | *1^st^ Tertile*  n =281 | *2^nd^ Tertile*  n = 327 | *3^rd^ Tertile*  n = 304 | *1^st^ Tertile*  n = 280 | *2^nd^ Tertile*  n = 234 | *3^rd^ Tertile*  n = 274 | p |
| Vitamin A (I.U./day) | 3985.10 (3418.84) | 5452.65 (4446.41) | 3667.35 (2749.39) | 2879.09 (2480.78) | 5563.51 (4564.53) | 3684.77 (2247.37) | 2686.26 (1580.45) | <0.001 |
| Thiamine (mg/day) | 0.93 (0.39) | 1.09 (0.40) | 0.97 (0.42) | 0.89 (0.39) | 1.03 (0.36) | 0.83 (0.33) | 0.71 (0.29) | <0.001 |
| Riboflavin (mg/day) | 1.01 (0.44) | 1.19 (0.46) | 1.04 (0.46) | 0.96 (0.45) | 1.13 (0.41) | 0.92 (0.37) | 0.77 (0.33) | <0.001 |
| Niacin (mg/day) | 19.61 (18.10) | 23.96 (19.18) | 21.39 (17.88) | 23.33 (22.97) | 17.36 (14.14) | 15.97 (13.99) | 14.33 (15.56) | <0.001 |
| Vitamin C (mg/day) | 161.90 (93.15) | 216.98 (126.92) | 158.47 (74.32) | 118.90(54.86) | 211.87 (104.22) | 147.28 (54.43) | 118.24 (63.58) | <0.001 |
| Vitamin D (mg/day) | 13.84 (20.54) | 15.34 (28.93) | 13.49 (14.47) | 14.79 (23.20) | 13.17 (13.61) | 13.93 (19.75) | 12.27 (19.85) | 0.529 |
| Calcium (mg/day) | 635.32 (285.55) | 762.87 (306.97) | 634.16 (285.41) | 578.57 (276.37) | 734.93 (278.28) | 598.60 (247.42) | 496.02 (211.96) | <0.001 |
| Phosphorous (mg/day) | 1120.03 (475.56) | 1309.57 (491.49) | 1161.44 (483.95) | 1156.61 (542.60) | 1162.09 (409.98) | 1005.84 (379.18) | 887.50  (387.15) | <0.001 |
| Iron (mg/day) | 15.22 (.713) | 18.84 (9.06) | 15.81 (6.08) | 15.35 (7.26) | 15.90 (5.26) | 13.10 (4.90) | 11.69 (6.98) | <0.001 |
| Zinc (mg/day) | 9.31 (3.44) | 11.09 (3.58) | 10.09 (3.58) | 10.00 (3.68) | 9.23 (2.83) | 7.92 (2.52) | 7.06 (2.33) | <0.001 |
| Iodine (µg/day) | 3.38 (6.62) | 3.60 (7.17) | 2.75 (7.41) | 1.74 (6.08) | 4.82 (6.41) | 4.15 (6.58) | 3.51 (5.25) | <0.001 |
| Copper (µg/day) | 1.79 (9.62) | 2.57 (8.75) | 2.31 (8.01) | 2.54 (9.55) | 1.08 (6.46) | 0.53 (5.56) | 1.34 (15.73) | 0.056 |
| Saturated fatty acid (g/day) | 13.83 (7.00) | 15.02 (5.10) | 16.10 (6.48) | 17.68 (10.09) | 11.20 (4.16) | 11.28 (4.64) | 10.52 (5.61) | <0.001 |
| MUFA (g/day) | 21.17 (9.43) | 23.66 (8.11) | 25.43 (9.08) | 26.59 (10.89) | 18.48 (6.87) | 18.92 (7.50) | 18.45 (9.24) | <0.001 |
| PUFA (g/day) | 14.97 (6.49) | 16.36 (5.41) | 16.66 (5.91) | 16.96 (8.01) | 13.57 (4.92) | 13.14 (6.28) | 12.34 (6.27) | <0.001 |
| Cholesterol (g/day) | 197.86 (179.03) | 218.94 (114.59) | 231.81 (135.30) | 236.74 (151.20) | 162.81 (75.73) | 167.46 (92.64) | 155.02 (343.75) | <0.001 |
| Fibre (g/day) | 9.77 (5.09) | 13.26 (6.98) | 9.56 (3.88) | 7.88 (3.28) | 12.19 (5.64) | 8.45 (3.06) | 7.13 (3.06) | <0.001 |
| Total Isoflavone (mg/day) | 15.26 (19.63) | 22.79 (33.01) | 15.87 (17.82) | 11.29 (13.59) | 19.30 (17.16) | 12.85 (12.82) | 8.88 (9.94) | <0.001 |
| Magnesium (mg/day) | 399.29 (194.62) | 468.92 (204.75) | 395.04 (180.51) | 341.37 (140.92) | 483.29 (221.09) | 377.54 (186.05) | 327.32 (170.15) | <0.001 |
| Vitamin K (µg/day) | 303.02 (211.42) | 407.60 (257.38) | 293.80 (229.52) | 219.95 (134.17) | 407.13 (233.91) | 281.82 (160.73) | 209.59 (99.72) | <0.001 |
| Sodium (mg/day) | 1570.57 (1011.93) | 1843.15 (796.34) | 1737.35 (852.06) | 1723.90 (1373.91) | 1475.14 (1039.84) | 1362.60 (862.20) | 1198.68 (838.24) | <0.001 |
| Potassium (mg/day) | 3002.77 (1239.06) | 3974.98 (1182.76) | 3269.47 (1037.60) | 2684.30 (1085.22) | 3456.80 (1208.27) | 2632.79 (975.30) | 1890.38 (696.90) | <0.001 |
| **Macronutrients** (Mean(SD)) |  |  |  |  |  |  |  |  |
| Protein (g/day) | 79.72 (33.51) | 93.60 (32.27) | 87.88 (34.24) | 88.04 (39.12) | 76.54 (27.14) | 68.54 (25.23) | 59.28 (25.28) | <0.001 |
| Fat (g/day) | 59.31 (23.61) | 64.52 (18.46) | 67.93 (22.60) | 70.60 (28.49) | 50.73 (16.37) | 50.35 (19.00) | 47.62 (22.07) | <0.001 |
| Carbohydrate (g/day) | 264.07 (85.60) | 286.46 (79.82) | 285.51 (89.19) | 305.60 (93.41) | 242.28 (73.02) | 227.20 (65.03) | 223.19 (67.36) | <0.001 |

*ANOVA was used to compare between groups; Note: MUFA = Monounsaturated Fatty Acid, PUFA = Polyunsaturated Fatty Acid*
